# SOX2 knockdown with siRNA reverses cisplatin resistance in NSCLC by regulating APE1 signaling

**DOI:** 10.1007/s12032-021-01626-3

**Published:** 2022-01-20

**Authors:** Tai-yu Chen, Ji Zhou, Peng-cheng Li, Chun-han Tang, Ke Xu, Tao Li, Tao Ren

**Affiliations:** 1 Clinical Medical College of Chengdu Medical College, Chengdu, 610500 China; 2 Health Management Centre, The First Affiliated Hospital, Chengdu Medical College, 278 Xindu St, Chengdu, 610500 Sichuan China; 3 Oncology Department, Clinical Medical College and The First Affiliated Hospital of Chengdu Medical College, 278 Baoguang St, Xindu Distr, Chengdu, 610500 Sichuan China; 4 Department of Radiation Oncology, School of Medicine, Sichuan Cancer Hospital & Institute, and Sichuan Cancer Center, University of Electronic Science and Technology of China, 55, 4th Section of Renmin South Road, Chengdu, 610041 Sichuan China

**Keywords:** Non-small cell lung cancer, DNA repair, Chemotherapy, Apoptosis

## Abstract

SOX2 is related to drug resistance in many types of cancer, including lung cancer. Herein, we investigated the role of SOX2 and its regulatory signaling in cisplatin-treated non-small-cell lung cancer (NSCLC). The effects of SOX2 on cell viability, proliferation, and apoptosis were evaluated in vitro. Western blotting, real-time quantitative PCR, immunohistochemistry, and luciferase reporter assays were used to investigate the underlying mechanism. Kaplan–Meier survival analysis and the log-rank test were used to assess the relationship between SOX2 expression and patient survival. A549/CDDP cells had marked resistance to cisplatin and stronger colony formation ability than A549 cells. The expression of SOX2 protein or mRNA in A549/CDDP was higher than that in A549. Knockdown of SOX2 in A549/CDDP-induced apoptosis by inhibiting colony formation and decreasing viability, but overexpression of SOX2 reversed these effects. Interestingly, Genomatix software predicted that the APE1 promoter has some SOX2 binding sites, while the SOX2 promoter has no APE1 binding sites. Furthermore, luciferase reporter assays proved that SOX2 could bind the promoter of APE1 in 293T cells. We further verified that SOX2 expression was not affected by shAPE1 in A549/CDDP. As expected, colony formation was obviously inhibited and apoptosis was strongly enhanced in A549/CDDP treated with SOX2 siSOX2 alone or combined with CDDP compared with control cells. Meaningfully, patients with low expression of SOX2, and even including its regulating APE1, survived longer than those with high expression of SOX2, and APE1. siSOX2 overcomes cisplatin resistance by regulating APE1 signaling, providing a new target for overcoming cisplatin resistance in NSCLC.

## Introduction

Drug resistance, whether intrinsic or acquired, is one of the most common challenges in cancer and facilitates tumor recurrence and disease progression [1]. Although platinum-based regimens are a cornerstone for the broad treatment of patients with non-small-cell lung cancer (NSCLC), the long-term survival of these patients remains poor largely due to the emergence of resistance prior to and during the course of treatment [2–4]. Thus, it is urgent to develop strategies that act on the cisplatin-resistant subpopulation of NSCLC and reverse the resistance of these tumor cells.

The transcription factor sex determining region Y-box 2 (SOX2, also called SRY) is a member of the high-mobility group (HMG) protein family related to SOX region Y and has an important role in the growth and development of mammals [5]. Sox2 plays a key role in various phases of embryonic development, including cell fate and differentiation. In particular, SOX2 knockout in the zygote results in early embryonic lethality [6]. Recently, emerging data have shown that aberrant SOX2 expression is also associated with various types of cancer, including lung cancer [7]. SOX2 is associated with disease progression, metastasis, and relapse in some cancer patients [5, 7–9]. Further, SOX2 may be involved in cancer stemness, as high SOX2 expression facilitates drug resistance [10]. Notably, SOX2 overexpression decreases cisplatin-induced cell apoptosis in lung cancer, while inhibition of SOX2 enhances cisplatin toxicity and promotes apoptosis in A549 and A549/CDDP cells [11], suggesting that SOX2 is a vital regulator of cisplatin resistance in NSCLC. A recent report found that SOX2 mediates cisplatin resistance in small cell lung cancer with downregulated expression of hsa-miR-340-5p. However, consensus SOX2 downstream targets that bear a tumorigenic function have not yet been fully clarified [5, 12]. Thus, further studies should be conducted to explore whether other molecules affect SOX2 in cisplatin-resistant lung cancer cells.

Notably, the Genomatix online platform for target prediction found that the promoter of APE1, a base-excision repair (BER) protein, has some binding sites for SOX2. Cisplatin treatment mostly induces DNA damage in cancer cells [13]. Upon recognition of a damaged base by specific glycosylases, apurinic/apyrimidinic endonuclease 1 (APE1, APEX, also known as REF-1) cleaves the newly generated abasic site, allowing DNA repair. APE1 is the key rate-limiting enzyme in the BER pathway [14]. The pivotal role of APE1 has been proven, as it has functions in cellular viability and embryonic development, and it also has DNA repair activity and other recently characterized noncanonical functions. In fact, APE1 also plays a key role as a redox effector on many transcription factors, such as NF-κB, HIF-1α, STAT-3, PAX8, AP-1 and p53, mediating pivotal genes involved in tumor progression or resistance [15]. Moreover, APE1 is abnormally highly expressed and results in a poor prognosis in lung cancer patients [16]. Our data showed that APE1 inhibition can enhance apoptosis in A549 or A549/CDDP cells via APE1-p53-LC3 complex assembly [3, 17]. Therefore, we wanted to know whether and how SOX2 plays a regulatory role in the APE1 protein.

In this report, our data showed that SOX2 expression was higher in A549/CDDP cells than in parental ADP cells and that NCI-H460 cells with lentivirus-mediated exogenous overexpression of SOX2 (OE-SOX2) had stronger colony formation and cellular viability and lower apoptosis rates than the corresponding control cells. In addition, SOX2 small-interfering siRNA (siSOX2)-mediated knockdown overcame cisplatin resistance in NSCLC by regulating APE1 signaling, providing a new therapeutic target to overcome cisplatin resistance in NSCLC patients.

## Materials and methods

### Cell lines and cell culture

The human lung cancer cell line A549 was obtained from the Chinese Academy of Science Cell Bank, and all cells were cultured in RPMI 1640 medium supplemented with 10% heat-inactivated fetal bovine serum (FBS). The cells were maintained in a humidified atmosphere containing 5% CO_2_ at 37 °C.

### Colony formation assays

A549 cells and A549/CDDP cells in good growth conditions were digested with trypsin, and the cell suspension was sufficiently resuspended to disperse the cells. Five hundred cells/dish were inoculated in 6-cm dishes, and each group was inoculated with 3 wells and cultured in an incubator for 13 days. The formation of clones was observed under a low-power microscope, and the culture was terminated if the number of cells per colony was greater than 50. The supernatant was discarded, and the cells were gently washed with room temperature PBS twice. Then, 4% paraformaldehyde was added to fix the cells at room temperature for 30 min. The fixative was removed, and then 0.2% crystal violet was added for staining at room temperature in the dark for 30 min. The crystal violet was discarded, the cells were gently washed with PBS once or twice, and the cells were naturally air-dried. For colony counting, the air-dried colony formation plate was placed on white paper painted with a grid, and the colonies were counted with the naked eye. The experiment was repeated 3 times.

### Western blotting

The cells were lysed in RIPA buffer with protease inhibitors and centrifuged at 12,000 rpm for 15 min, and then the supernatant was collected. The protein lysates (30 μg) were separated by sodium dodecyl sulfate-PAGE and transferred to a polyvinylidene difluoride membrane. After blocking, the membrane was incubated with anti-human polyclonal antibodies against SOX2 and then with a secondary antibody (mouse IgG HRP-conjugated antibody). Visualization was performed using the Bio-Rad ChemiDoc XRS system, and the blot bands were analyzed using Image Lab 3.0.

### Real-time quantitative fluorescence PCR (qRT-PCR)

The cells were collected, and total RNA was extracted with a total RNA extraction kit; the extracts were stored at − 80 °C. Then, cDNA was synthesized by reverse transcription according to the instructions of the reverse transcription PCR kit. The concentration and purity of the synthesized cDNA were detected. SOX2 mRNA was synthesized by cDNA reaction according to the real-time PCR instructions. The reaction procedure was as follows: 95 ℃ for 1 min; 95 ℃ 40 s, 58 ℃ 40 s, 72 ℃ 45 s, 35 cycles; 72 ℃ for 10 min.

### Lentivirus transfection

The cells were inoculated at 1 × 10^5^ cells/well in a 6-well plate. After the cells completely attached to the plate, the virus volume for virus infection was calculated according to the formula: (MOI × number of cells)/virus titer. After staining for 12 h, the medium was replaced with normal medium for further culture after 24 h. The number of cells with fluorescence expression was higher at 72 h, and the infection efficiency was observed by fluorescence microscopy. The rate was approximately 80%, and the cell growth state was good. Then, we added puromycin to the medium for screening for 48 h, and fluorescence was detected. The cells were observed and identified under a light microscope, expanded, collected and frozen for later use.

### Cell counting Kit-8 (CCK-8) assays

The cells were inoculated in 96-well plates at 5000 cells/well, and cisplatin was added at different concentrations for 24 h and 48 h. Medium containing 10% CCK-8 detection reagent was added to each well and incubated at 37 °C for 2 h. The absorbance at 450 nm was detected, and the survival rate of cells was calculated. The experiment was repeated 3 times in each group.

### Quantification of cellular apoptosis

Cell apoptosis was evaluated using an annexin V-PE apoptosis detection kit as previously described. Briefly, the cells were collected after treatment and resuspended in 1 × binding buffer with 5 μL of annexin V-PE, 5 μL of 7-amino-actinomycin D (7-AAD) and 1 × 10^5^ cells/mL in a total volume of 150 μL. The cells were gently mixed and incubated in the dark for 15 min at room temperature. Binding buffer (100 μL) was then added to each tube, and the number of apoptotic cells was quantified using flow cytometry. Ten thousand events were collected for analysis.

### Dual-luciferase report assay

The wild-type and mutant-type dual-luciferase reporter vectors of APE1 were transfected into SOX2-OE 293T cells and control cells. After transfection, the cells were cultured for 48 h, and then the cells were collected and lysed at room temperature for 20 min. The supernatant was collected by centrifugation and stored at − 20 °C or directly added to the luciferase substrate. The relative firefly luciferase activity was calculated using the sea kidney luciferase activity as an internal reference.

### Patients and clinical specimens

A total of 45 patients were enrolled in this study between January 2018 and February 2019. All patients were from the First Affiliated Hospital of Chengdu Medical College. The local ethics committee approved the study. Postoperative disease staging was performed according to the American Joint Committee on Cancer (AJCC) (8th edition), from stage IIA to stage IIIB. All patients received radical surgery without preoperative chemotherapy or radiotherapy and at least four cycles of adjuvant chemotherapy with cisplatin. Postoperative tissue specimens were fixed in 10% neutral formalin solution (pH 7.2) at room temperature for 24–48 h and then made into wax blocks for use.

### Immunohistochemistry (IHC)

The expressions of SOX2 and APE1 protein were analyzed by IHC [18]. Paraffin-embedded tumor sections were incubated overnight with mouse anti-human SOX2 monoclonal antibody (1:2000) or APE1 antibody (1:1000) and then incubated with goat anti-mouse secondary antibody. The antigen–antibody complex was observed by incubation with a 3,3-diaminobenzidine (DAB) substrate and staining with diluted Harris hematoxylin. The cell number score was also assessed. Five fields of 200 cells/field were randomly selected under high magnification (×40).

### Statistical analysis

GraphPad prism 8 were used to analyze the data. The data are expressed as the mean ± standard deviation (SD). One-way analysis of variance (ANOVA) followed by Tukey’s multiple comparison procedure was used for comparisons of multiple groups. Survival curves were plotted using the Kaplan–Meier method, and significant differences between life expectances were determined using log-rank tests. And *P* < 0.05 indicated a significant difference.

## Results

### SOX2 is highly expressed in A549/CDDP cisplatin-resistant cells

**Fig. 1:**
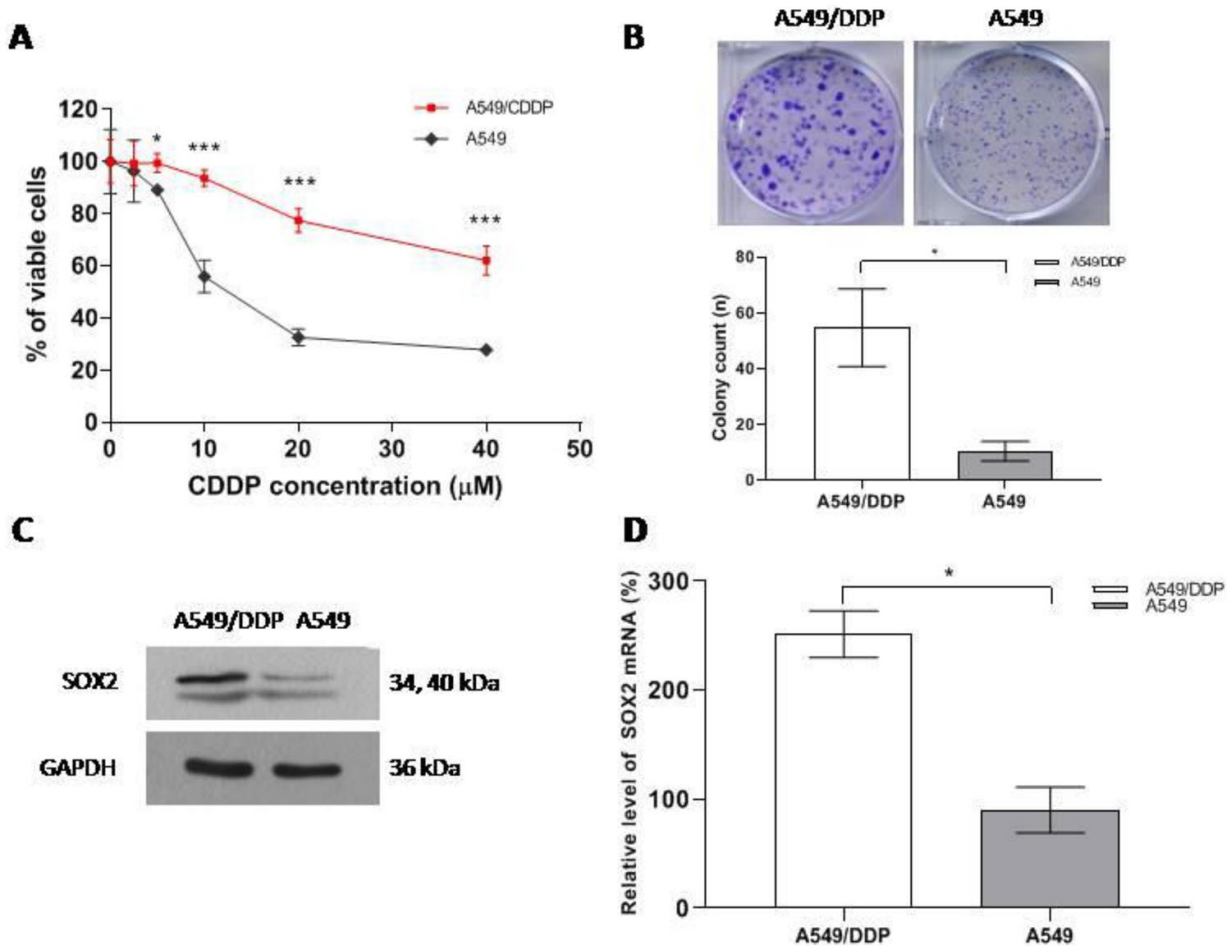
SOX2 is highly expressed in A549/CDDP cisplatin-resistant cells. **A** Cellular viabilities from the CCK-8 assay. **B** Cellular proliferation from colony formation assays. **C** SOX2 expression via western blotting. **D** SOX2 mRNA level from qRT-PCR. * < 0.05

To investigate the underlying mechanism of cisplatin resistance and the related signaling molecules, we successfully cultivated A549/CDDP cells with acquired cisplatin resistance and the parental A549 cells [3]. Compared with A549 cells, A549/CDDP cells had stronger cisplatin resistance and colony formation ability (Fig. 1A, B). SOX2, a stemness biomarker, is related to drug resistance in many cancers [19, 20]. First, we assessed the protein and mRNA expression levels of SOX2 between A549/CDDP and A549 cells via western blotting and qRT-PCR, respectively. As shown in Fig. 1C, D, SOX2 expression was higher in A549/CDDP cells than in A549 cells at both the protein and mRNA levels. All these data suggest that cisplatin resistance may be correlated with SOX2 overexpression in NSCLC.

### Inhibition of SOX2 decreases colony formation and viability and increases apoptosis in A549/CDDP cells

**Fig. 2:**
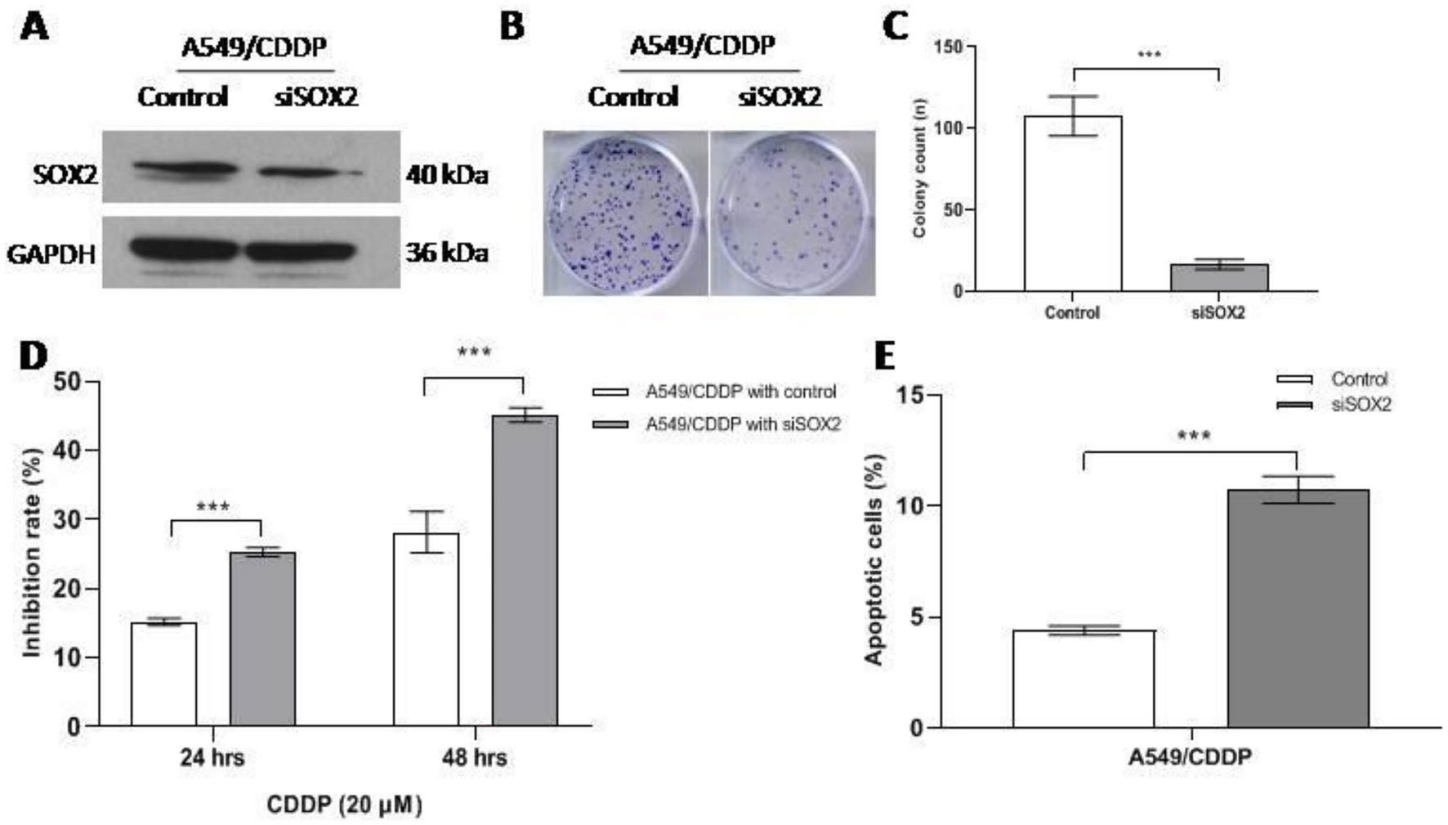
Inhibition of SOX2 decreases A549/CDDP colony formation, viability and increases apoptosis. **A** SOX2 expression in A549/CDDP treated with siSOX2 via western blotting. **B** and **C** The difference in cellular colony formation between siSOX2 group and control group. **D** Inhibition rate of A549/CDDP treated with siSOX2 via CCK-8 assay. **E** Apoptosis of A549/CDDP cells with SOX2 siRNA treatment via annexin V-PE/7-AAD. *** < 0.0001

SOX2 is highly expressed in A549/CDDP cells. We wondered what would happen if we knocked down SOX2 by transducing the cells with lentivirus expressing siSOX2. Through colony formation assays, we found that A549/CDDP cells in the siSOX2 group formed fewer colonies than those in the control group (Fig. 2A–C). Then, we carried out CCK-8 assays to analyze cellular viability. The data showed that, upon cisplatin treatment, compared with the control group, the siSOX2 group of A549/CDDP cells had lower viability at both 24 h and 48 h (Fig. 2D). Furthermore, we found that apoptosis was obviously increased in the siRNA treatment group compared with the control group (Fig. 2E). This suggests that suppression of SOX2 decreases colony formation and viability and increases apoptosis in A549/CDDP cells.

### Lentivirus-mediated overexpression of SOX2 increases colony formation and viability and inhibits apoptosis in A549 cells

**Fig. 3:**
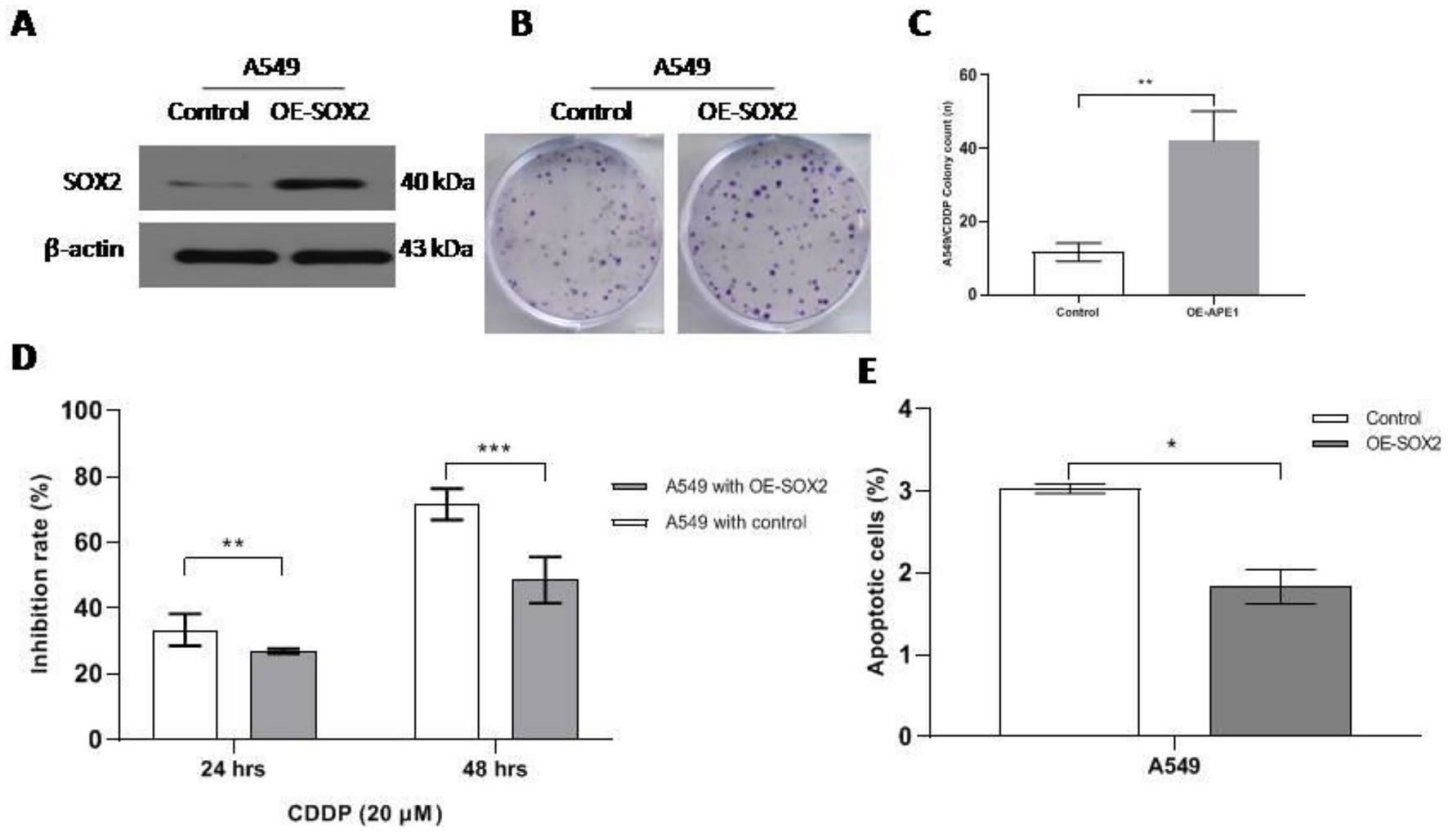
Lentivirus-mediated overexpression of SOX2 promotes colony formation and viability and inhibits apoptosis in A549 cells. **A** SOX2 expression in A549 cells with OE-SOX2 via western blotting. **B** and **C** The difference in cellular colony formation between the siSOX2 group and the control group. **D** Inhibition rate of A549cells with OE-SOX2 via CCK-8 assay. **E** Apoptosis of A549 cells with OE-SOX2 treatment via annexin V PE/7-AAD. * < 0.05, ** = 0.0016, *** < 0.0001

SOX2 overexpression induces tumor progression, recurrence and metastasis in many cancers, including NSCLC [7]. We carried out lentivirus-mediated overexpression of SOX2 (OE-SOX2) in A549 cells. As expected, compared with the control group, the OE-SOX2 group of A549 cells had increased colony formation ability (Fig. 3A–C) and cellular viability with cisplatin treatment (Fig. 3D). Furthermore, flow cytometry was used to evaluate apoptosis, and A549 cells in the OE-SOX2 group had a lower apoptosis rate than those in the control group (Fig. 3E). Together, these findings show that high SOX2 expression can inhibit apoptosis by enhancing cell proliferation and viability.

### SOX2 regulates APE1 signaling, which is directly involved in cisplatin resistance in NSCLC

**Fig. 4:**
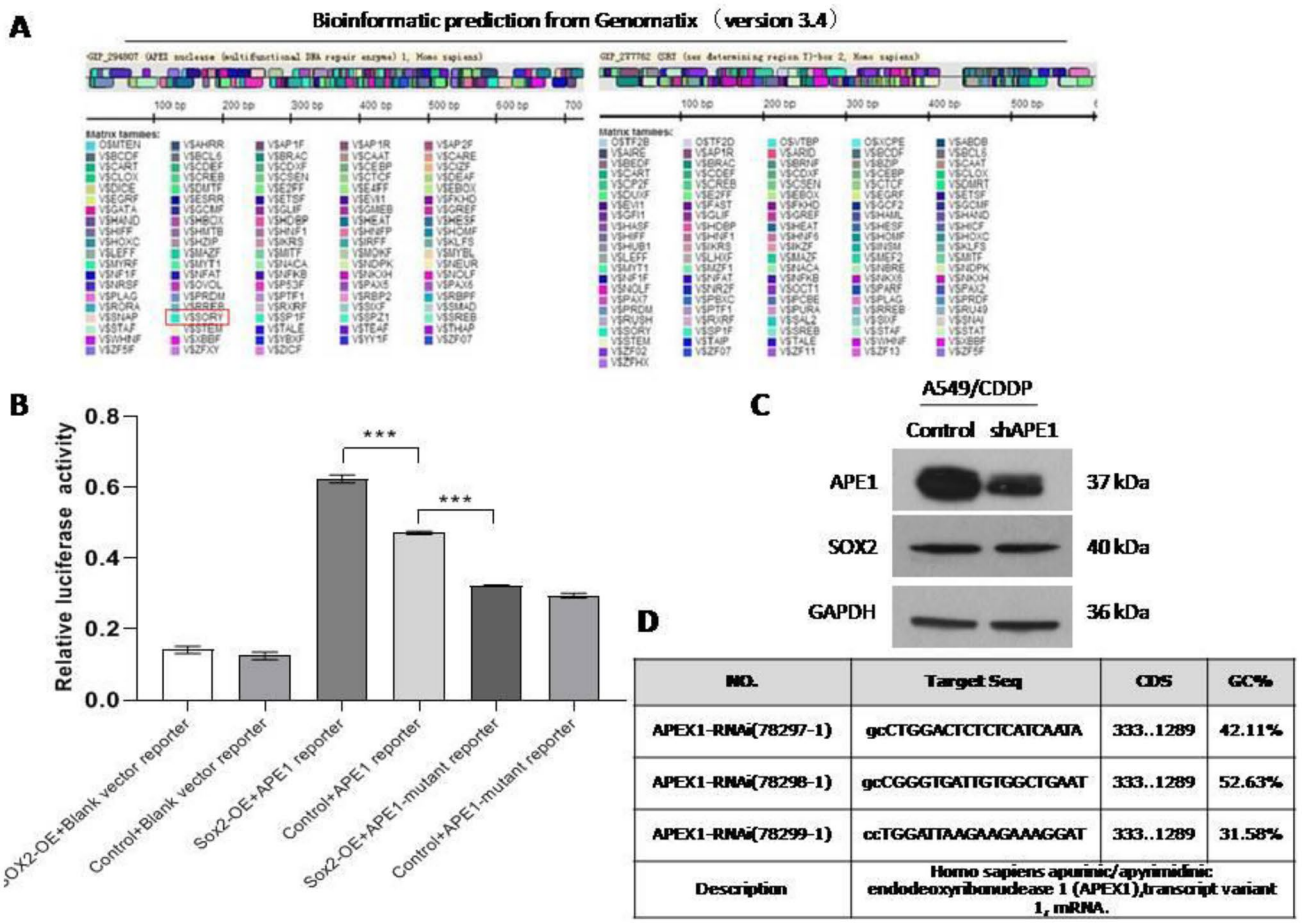
SOX2 regulates APE1 signaling, which is directly involved in cisplatin resistance in NSCLC. **A** Bioinformatic predictions from Genomatix. **B** The data from dual-luciferase reporter assays in 293T cells expressing APE1 wild-type or mutant reporters, combined with overexpression of SOX2 or endogenous SOX2. **C** APE1 or SOX2 expression in A549/CDDP cells treated with shAPE1 or control via western blotting. **D** Sequences of shRNA targeting APE1 successfully designed. *** < 0.0001

Emerging data have revealed that SOX2 is involved in many molecular events and has a key role in cancer [5]. We first used Genomatix (version 3.4) to predict the possible mechanism by which SOX2 regulates cisplatin resistance. Interestingly, the APE1 promoter had some SOX2 binding sites, but the SOX2 promoter had no APE1 binding sites (Fig. 4A). Next, we used dual-luciferase reporter assays to verify whether APE1 is a direct target of SOX2 in 293T cells. As expected, OE-SOX2 dramatically enhanced the luciferase activities of wild-type APE1 reporters, while it markedly decreased those of the mutant reporter and blank vector reporter (Fig. 4B). Moreover, we assessed whether APE1 affects SOX2 expression. As shown in Fig. 4C, SOX2 protein expression hardly changed in A549/CDDP cells with lentivirus-mediated transduction of a small hairpin RNA targeting APE1 (shAPE1) that we successfully designed (Fig. 4D). These data suggest that APE1 is a direct target of SOX2 involved in cisplatin resistance in NSCLC.

### siSOX2 reverses cisplatin resistance in A549/CDDP cells

**Fig. 5:**
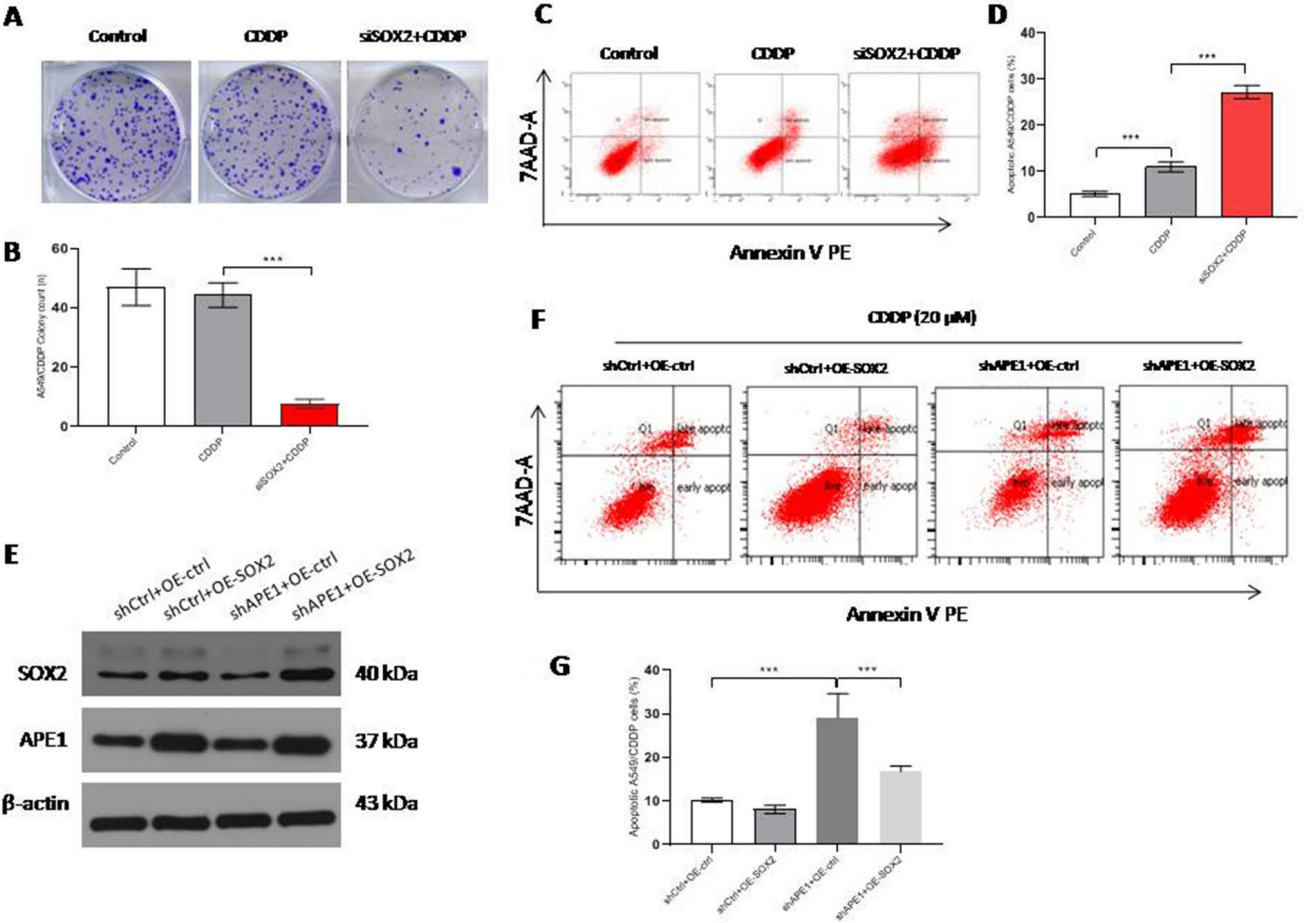
siSOX2 reverses cisplatin resistance in A549/CDDP cells. **A** and **B** Colony formation in A549/CDDP cells treated with control, cisplatin, or cisplatin combined with siSOX2. **C** and **D** Apoptosis in A549/CDDP cells treated with control, cisplatin, or cisplatin combined with siSOX2. **E** Western blot assays showed the expressions of SOX2 and APE1 in A549/CDDP cells treated with SOX2 overexpression and/or shAPE1. **F** and **G** The sensitivity of cells to cisplatin in a context of SOX2 overexpression and/or APE1 silencing in A549/CDDP cells via flow cytometry. *** < 0.0001

Next, we wanted to see whether siSOX2 could reverse cisplatin resistance in A549/CDDP cells. First, we used a colony formation assay to test the effect on cell proliferation ability. Inspiringly, the number of colonies formed in the combined siRNA and 20 μM cisplatin treatment group was less than that in the control or siRNA treatment group (Fig. 5A, B). More importantly, apoptosis was strongly enhanced in the combined siRNA and 20 μM cisplatin treatment group compared with the control or cisplatin treatment group (Fig. 5C, D). Furthermore, we wanted to see the sensitivity of cells to cisplatin in a context of SOX2 overexpression and APE1 silencing, the data showed that APE1 silencing obviously increased the apoptosis of A549/CDDP cells treated with 20 μM cisplatin, and interestingly SOX2 overexpression reversed the effect from APE1 silencing in A549/CDDP treated with 20 μM cisplatin (Fig. 5E–G). Another important thing is about that we found APE1 expression level was significantly increased in A549/CDDP cells treated with SOX2 overexpression via using western blot (Fig. 5E), which meant SOX2 affecting APE1 expression via binding the promoter of APE1(Fig. 4A–B).

All these data suggest that siSOX2 rescues resistance to cisplatin in NSCLC, which makes SOX2 a new target for sensitizing NSCLC tumor cells to cisplatin.

### NSCLC patients with SOX2 low expression had good survival

**Fig. 6:**
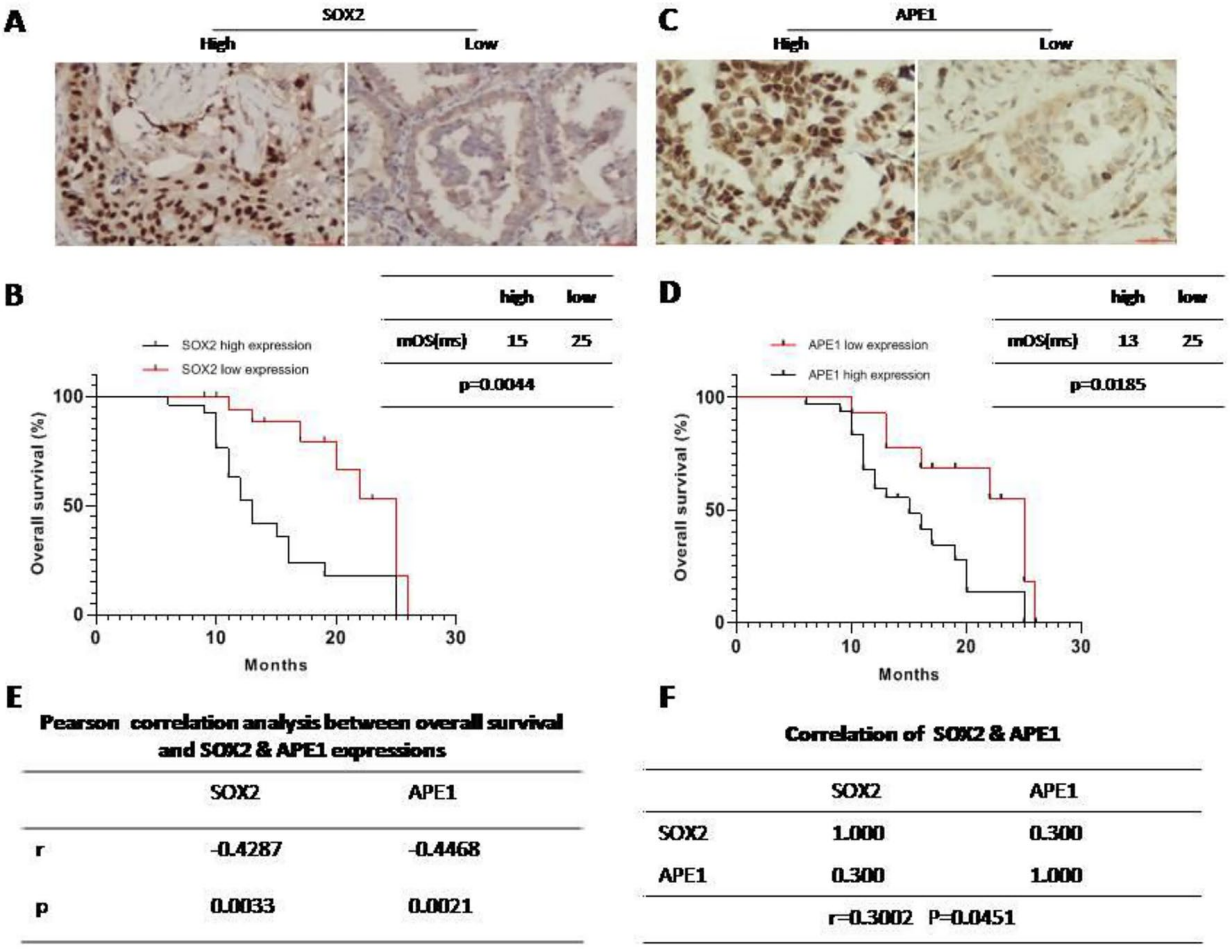
NSCLC patients with SOX2 overexpression had good survival. **A** SOX2 expression in lung adenocarcinoma via IHC. **B** Kaplan–Meier survival analysis and log-rank test on the basis of SOX2 level for 45 patients with lung adenocarcinoma. **C** APE1 in lung adenocarcinoma via IHC. **D** Kaplan–Meier survival analysis and log-rank test on the basis of APE1 level for 45 patients with lung adenocarcinoma. **E** and **F** Pearson correlation analysis between overall survival and SOX2 & APE1 expression. *mOS* means mean overall survival, *ms* means months

We also collected 45 samples from stage IIA and IIIA-IIIB NSCLC patients who underwent surgery in our hospital from January 2018 to December 2019. The follow-up time was 15 months. These patients received at least four cycles of adjuvant chemotherapy with a cisplatin regimen. We further investigated the expression of SOX2 by IHC. As shown in Fig. 6A, SOX2 expression was variable; 62.2% (28/45) of samples had high expression, and 37.8% (17/45) of samples had low expression. Next, we applied Kaplan–Meier survival analysis and the log-rank test to analyze whether SOX2 expression affects NSCLC patient survival. The results showed that patients with low SOX2 expression survived longer than patients with high SOX2 expression; the median overall survival times were 25 months vs 15 months, respectively (Fig. 6B). We have also checked the APE1 expressions in samples tissues and found that 68.9% (31/45) of samples had high expression, and 31.1% (14/45) of samples had low expression (Fig. 6C). And patients with high APE1 expression survived shorter, compared with APE1 low expression. The median overall survival times were 13 months vs 25 months, respectively (Fig. 6D). Furthermore, we did pearson correlation analysis between overall survival and SOX2 and APE1 expression, found that long survival was negative correlation with SOX2 or APE1 expression (Fig. 6E), and APE1 was also apparently correlated with SOX2 (Fig. 6F). Taken together, these findings indicate that SOX2 expression, including its regulating protein APE1, is correlated with NSCLC patient prognosis and has bright anticancer prospects.

## Discussion

With the development and application of targeted therapy and immunotherapy [21], the use of traditional chemotherapy is increasingly restricted and denounced in NSCLC. Even so, platinum is still administered widely for the treatment of NSCLC [22, 23]. However, long-term survival in patients with NSCLC remains poor largely due to the emergence of resistance prior to and during the course of treatment [2–4]. Thus, the exploration of novel driver oncogenes is urgently needed in order to identify targets for reversing drug resistance in NSCLC.

In this report, we found that the SOX2 protein or mRNA expression in A549/CDDP cells was higher than that in A549 cells [20]. Knockdown of SOX2 in A549/CDDP cells induced apoptosis by inhibiting colony formation and decreasing cell viability [24], but overexpression of SOX2 reversed these effects [24]. This suggests that SOX2 promotes cell growth in NSCLC, but Koki Takeda et al. thought that SOX2 may suppress cell proliferation in colorectal cancer [25]. SOX2, as a putative marker of undifferentiated cells [25, 26], has additional roles in adult tissue homeostasis and regeneration [27]. Recently, aberrant expression of SOX2 has been demonstrated in various types of cancers, including NSCLC [19, 24, 25, 28, 29]. Additionally, studies found that SOX2 regulates the stem cell properties and drug resistance of cancer cells [10], which was consistent with our previous findings for the role of SOX2 in NSCLC A549/CDDP cells published on Chinese journal. The mechanisms of resistance to cisplatin among different cell types are different and complicated [20]. Notably, SOX2 is involved in cisplatin resistance events [20, 30, 31]. Emerging downstream targets regulating SOX2, such as cyclin D1, Nanog, SKIL, and PI3K, have been found [5, 32, 33]. However, to date, the downstream targets are not fully understood. On the other hand, studies aimed at functional depletion, for example, by siRNA knockdown, are difficult to translate into clinical settings. Alternatively, the identification of upstream or downstream regulators of SOX2 that are easier to target is of utmost importance [32].

Interestingly, Genomatix software [34] predicted that the APE1 promoter has some SOX2 binding sites but that the SOX2 promoter has no APE1 binding sites. Further, APE1 was highly expressed in A549/CDDP cells compared with A549 cells, suggesting that it may benefit cisplatin resistance [35]. APE1 expression was decreased or increased with knockdown or overexpression of SOX2 in NCI-H460 cells, respectively. We also found that SOX2 expression was not affected by shAPE1 in A549/CDDP cells in vitro. Furthermore, luciferase reporter assays further proved that SOX2 could bind the promoter of APE1 in 293T cells. All these data suggest that SOX2 induces cisplatin resistance in NSCLC by regulating APE1. In addition, this is the first report indicating that APE1 is a direct downstream target of SOX2.

As expected, colony formation was obviously inhibited and apoptosis was strongly enhanced in A549/CDDP cells treated with siSOX2 alone or combined with CDDP compared with the control cells [11]. Finally, we found that SOX2 expression was different in 45 advanced NSCLC patients and that patients with low expression of SOX2 survived longer than those with high expression of SOX2, which was consistent with some previous report [10]; Some studies reported that SOX2 is a predictor of poor survival in upper tract urothelial carcinoma, breast cancer and small cell lung cancer [36–38].

In conclusion, we found that SOX2 was overexpressed in A549/CDDP cells with acquired resistance versus parental A549 cells. Further study revealed that siSOX2 overcomes cisplatin resistance in NSCLC by regulating APE1 signaling, providing a new therapeutic target for NSCLC patients.

## Data Availability

The datasets used in the current study are available from the corresponding author on reasonable request.
